# Predicting health-related quality of life (EQ-5D-5 L) and capability wellbeing (ICECAP-A) in the context of opiate dependence using routine clinical outcome measures: CORE-OM, LDQ and TOP

**DOI:** 10.1186/s12955-018-0926-7

**Published:** 2018-05-30

**Authors:** Jasmine Peak, Ilias Goranitis, Ed Day, Alex Copello, Nick Freemantle, Emma Frew

**Affiliations:** 1 Health Economics Unit, Institute of Applied Health Research, Public Health Building, University of Birmingham, B15 2TT, Birmingham, UK; 2 Research and Innovation Department, Birmingham & Solihull Mental Health NHS Foundation Trust, Birmingham, UK; 3 Addictions Department, Institute of Psychiatry, Psychology & Neuroscience, King’s College London, London, UK; 4 School of Psychology, University of Birmingham, Birmingham, UK; 5 Department of Primary Care and Population Health, University College London, London, UK; 6 Melbourne School of Population and Global Health, University of Melbourne, Melbourne, Australia

**Keywords:** ICECAP, EQ-5D, Mapping, Addiction, Mental health, Preference-based measures, Condition-specific measures, Economic evaluation

## Abstract

**Background:**

Economic evaluation normally requires information to be collected on outcome improvement using utility values. This is often not collected during the treatment of substance use disorders making cost-effectiveness evaluations of therapy difficult. One potential solution is the use of mapping to generate utility values from clinical measures. This study develops and evaluates mapping algorithms that could be used to predict the EuroQol-5D (EQ-5D-5 L) and the ICEpop CAPability measure for Adults (ICECAP-A) from the three commonly used clinical measures; the CORE-OM, the LDQ and the TOP measures.

**Methods:**

Models were estimated using pilot trial data of heroin users in opiate substitution treatment. In the trial the EQ-5D-5 L, ICECAP-A, CORE-OM, LDQ and TOP were administered at baseline, three and twelve month time intervals. Mapping was conducted using estimation and validation datasets. The normal estimation dataset, which comprised of baseline sample data, used ordinary least squares (OLS) and tobit regression methods. Data from the baseline and three month time periods were combined to create a pooled estimation dataset. Cluster and mixed regression methods were used to map from this dataset. Predictive accuracy of the models was assessed using the root mean square error (RMSE) and the mean absolute error (MAE). Algorithms were validated using sample data from the follow-up time periods.

**Results:**

Mapping algorithms can be used to predict the ICECAP-A and the EQ-5D-5 L in the context of opiate dependence. Although both measures can be predicted, the ICECAP-A was better predicted by the clinical measures. There were no advantages of pooling the data. There were 6 chosen mapping algorithms, which had MAE scores ranging from 0.100 to 0.138 and RMSE scores ranging from 0.134 to 0.178.

**Conclusion:**

It is possible to predict the scores of the ICECAP-A and the EQ-5D-5 L with the use of mapping. In the context of opiate dependence, these algorithms provide the possibility of generating utility values from clinical measures and thus enabling economic evaluation of alternative therapy options.

**Trial registration:**

ISRCTN22608399. Date of registration: 27/04/2012. Date of first randomisation: 14/08/2012.

**Electronic supplementary material:**

The online version of this article (10.1186/s12955-018-0926-7) contains supplementary material, which is available to authorized users.

## Background

In many healthcare systems around the world, resources are scarce and the demand for healthcare outweighs supply. This scarcity warrants the need for economic evaluation to aid decision makers with information about the most efficient use of resources in order to maximise the health gained for every unit of currency spent. Within the UK, and in many other country jurisdictions, the most common approach to economic evaluation is the cost-utility analysis. The outcomes of a cost-utility analysis are expressed in quality-adjusted life years (QALYs) [1]. QALYs take into account both quality and length of life and offer a commensurate unit that allows comparisons of cost-effectiveness across different disease areas and interventions [2]. To measure QALYs, generic preference-based measures of health-related quality of life (HRQOL) are required [3] to capture a broad construct of health through key dimensions that are known to affect quality of life [4]. Commonly, the EuroQol-5D (EQ-5D-5 L) measure is used in economic evaluations to estimate QALYs [5]. The EQ-5D-5 L describes HRQOL through the dimensions of mobility, self-care, usual activities, pain and discomfort, and anxiety and depression.

When considering substance use disorders, the broader concept of wellbeing is considered more appropriate to reflect the clinical and policy objectives [6]. Drug dependence undermines an individual’s capability [6], and this disempowerment is largely overlooked in the health economics of addiction-related interventions as a result of the narrow definition of HRQOL. Until recently, it was hard to define and quantify wellbeing for the purposes of an economic evaluation. Amartya Sen’s work on capabilities allowed for a conceptualization of wellbeing through human functionings (what an individual ‘does’) and capabilities (the ability of the individual to exercise a functioning) [7]. The development of the ICEpop CAPability measure for Adults (ICECAP-A) based on Amartya Sen’s capability approach, means that wellbeing can now be measured in a way that is compatible for use in economic evaluations [8]. The ICECAP-A measures capability wellbeing through the dimensions of stability, enjoyment, achievement, attachment and autonomy. Both EQ-5D-5 L and ICECAP-A were found to have the appropriate construct validity within the addiction context, but ICECAP-A appeared to be significantly more responsive to changes of key clinical indicators [9].

In order to reduce the burden of assessment on patients and to help acquire data for clinical means, particularly in the context of substance use disorder, studies tend to rely only on clinical context-specific measures. For substance use disorders, measures that are commonly used to assess the level and impact of dependence and assess the effectiveness of a treatment are the Clinical Outcomes in Routine Evaluation - Outcome Measure (CORE-OM), the Leeds Dependence Questionnaire (LDQ), and the Treatment Outcomes Profile (TOP). The CORE-OM and the TOP are instruments that are used to assess the treatment outcome, whilst the LDQ is used to assess the level of dependence at the time of assessment [10, 11]. These measures, however, are unsuitable for use within health economic evaluations [12]. To enable information from these measures to be used in economic evaluations, a process called ‘mapping’ can be applied [13]. Mapping, quantifies the relationship between different measures using appropriate statistical techniques [14, 15], and allows for the estimation of HRQOL and wellbeing for use in economic evaluations using data collected from routine clinical measures.

This study aims to map three clinical instruments that are often used in the routine care of addiction and opiate dependence (CORE-OM, LDQ, and TOP) onto the EQ-5D-5 L and ICECAP-A measures, generating algorithms that can be used in future studies to aid reimbursement decisions in the absence of information related to EQ-5D-5 L and ICECAP-A. With these mapping algorithms, data from the three clinical measures can be translated into health and capability scores for use in an economic evaluation. The mapping algorithms were developed using data from a pilot randomised control trial (RCT) that sought to explore the effectiveness of two psychosocial interventions for heroin users receiving opiate substitution treatment (OST) in England [10]. This is the first study to develop mapping algorithms from routine clinical outcome measures used in addiction therapy onto the EQ-5D-5 L and ICECAP-A.

## Methods

The study uses data collected as part of a pilot randomized controlled trial designed to investigate the clinical and cost-effectiveness of two psychological interventions delivered in addition to the usual care of individuals who had been receiving opiate substitution treatment for more than one year [10]. All trial participants met the ICD-10 criteria for opioid dependence and were recruited if they were in opiate substitution treatment with methadone or buprenorphine for more than a year but still reported heroin use during the last month. The only exclusion criteria were having a physical or mental health condition that prevented engagement in the psychosocial intervention, or an imminent period of imprisonment. A number of client outcomes, including mental health (CORE-OM), substance dependence (LDQ), physical and psychological health (TOP), health-related quality of life (EQ-5D-5 L), and capability wellbeing (ICECAP-A) were assessed at baseline, 3 months and 12 months post-randomisation. These outcome measures are described in detail below. The trial was conducted by three community drug teams in England. All trial participants provided written informed consent, and ethical approval was obtained from the Black Country NHS Research Ethics Committee (reference: 12/WM/0046).

### Outcome measures

#### Clinical outcomes in routine evaluation - outcome measure (CORE-OM)

The CORE-OM is widely used to assess the mental health effects of psychological interventions [16]. It comprises 34 items across four main areas of focus: subjective wellbeing, symptoms, functioning and risk. A five point rating scale has been adopted, with 0 representing not at all, whilst 4 represents all the time [17]. A mean item score is commonly generated to allow an understanding of the level of psychological distress of an individual. The CORE-OM is a widely used measure that generates clinically meaningful information [17], and it has been regarded as acceptable, reliable, valid [16].

#### Leeds dependence questionnaire (LDQ)

The LDQ is used to identify an individual’s level of dependence on a variety of substances [18]. It features 10 items that are rated on a scale from 0 to 3; 0 representing never, whilst 3 represents nearly always. The questions are centered around substance use and frequency; asking about desires, how substances fit into daily routines and any future plans of taking substances [19]. The scores of all items are aggregated to indicate the overall level of dependence. This can vary between 0 and 30 with the cut-offs of 10 and 22 used to classify individual’s level of dependence into low, moderate, and severe. Raistrick et al. [18] describe a variety of features of LDQ that suggest it may be able to complement economic evaluation. The authors conclude that LDQ is understandable and sensitive to change in the level of dependency over time and across all substance dependencies.

#### Treatment outcomes profile (TOP)

The TOP is used to assess the change and progress in key areas of life for individuals who are being treated for drug or alcohol addiction [20]. It features 20 questions, reflecting the four domains of substance use- injecting risk behaviour, crime and health and social care functioning [21]. For this study, the health and social care functioning aspect, which included psychological health, physical health and the overall quality of life dimensions were of importance. These three dimensions are rated on a scale of 0 (which represents poor) to 20 (which represents good) [20].

#### EuroQol – 5 dimensions – 5 levels (EQ-5D-5 L)

The EQ-5D-5 L is an instrument that is used to measure health-related quality of life. It is a self-reported questionnaire that covers five dimensions of health; mobility, self-care, usual activities, pain and discomfort, and anxiety and depression. Participants select their functioning level from five options ranging from 1 to 5, with 1 representing that an individual has no functioning problems in a given dimension, whereas 5 represents severe problems with functioning [22]. An index of health-related quality of life is generated to illustrate an individual’s overall health status using a population tariff. This study used the English population tariff which was developed based on the time trade-off and discrete choice experiment methods [23]. The health index score ranges from − 0.281 to 1, with negative values representing health states worse than death, 0 representing the “dead” state, and 1 the “full health” state. The reliability and validity of the EQ-5D-5 L for use with the population of study in this investigation has already been published [9].

#### ICEpop CAPability measure for adults (ICECAP-A)

The ICECAP-A is a measure of capability wellbeing. It focuses on ability to function across five key dimensions of wellbeing. These are stability, enjoyment, achievement, attachment and autonomy. Participants select their capability level from four options ranging from 1 to 4, with 1 representing that an individual has limited capability in a given dimension, whereas 4 represents high levels of capability [8]. An index of capability wellbeing is generated that illustrates an individual’s overall capability levels from 0 to 1 using a UK population tariff developed based on the best-worst scaling method [24]. A score of 0 suggests that an individual has no capability, whilst 1 represents full capability [8]. The reliability and validity of the ICECAP-A for use with the population of study in this investigation has also been published [9].

### Estimation and validation datasets

A common approach across mapping studies is to split the dataset into an estimation dataset, where the mapping algorithm between the source outcome measures (CORE-OM, LDQ and TOP) and the target measures (EQ-5D-5 L and ICECAP-A) is first derived, and a validation dataset, where the predictive properties of the algorithm are tested [25]. Two approaches were used to determine the estimation and validation dataset. In the first approach, the estimation sample was developed from the baseline data and the validation sample from the 3 month follow-up data. In the second approach, the data from the baseline assessment and 3 month follow up were pooled in order to create a larger estimation sample, and the 12 month follow up data were then used for validation purposes, similar to other studies in the literature [26–28].

### Statistical analysis

Longworth and Rowen [25] highlight a variety of regression methods that are often used in mapping studies. The type of method employed depends on whether the prediction goal is for the overall index score or the dimension scores of a preference-based measure. Given that mapping onto an overall index score rather than dimension scores has been found to offer better predictive ability [29], this approach was adopted for the purposes of this study.

In the first mapping approach, ordinary least squares (OLS) and tobit regressions were used. The OLS regression approach is commonly used in mapping studies [25] and has been regarded as robust at predicting the mean index score of a preference-based measure [30]. The tobit regression is a censored regression method providing opportunity to limit predictions within the appropriate range of scores for EQ-5D-5 L (− 0.281 to 1) and ICECAP-A (0 to 1). Given that the OLS regression is likely to provide predictions beyond these ranges, these predictions were subsequently forced to the appropriate threshold value. This is.

a common approach in mapping studies [31–34]. In the second mapping approach, a cluster regression and a multilevel mixed effects regression at an individual level were used to account for within-subject dependence [35, 36].

A number of potential explanatory variables were available and these were explored incrementally and in line with the recommended methods guidance provided by Longworth and Rowen [25]. These included overall scores, dimension scores, quadratic terms for potentially nonlinear relationships, interaction terms, and patient characteristics (i.e. age and gender). All model specifications used to map from CORE-OM, LDQ and TOP onto the EQ-.

5D-5 L and ICECAP-A are shown in Table 1.

**Table 1:** Summary of the model specifications used when mapping from CORE-OM, LDQ, and TOP onto EQ-5D-5 L and ICECAP-A

Model	CORE-OM	LDQ	TOP
1	Mean score	Aggregate score	Overall quality of life score
2	Mean score; Mean score^2^	Aggregate score; Aggregate score^2^	Overall quality of life score; Physical and Psychological health status
3	Wellbeing; Symptoms; Functioning; Risk	Best model from above plus Age and Age^2^	Model 2 plus quadratic terms
4	Model 3 plus quadratic terms	Model 3 plus Gender	Model 3 plus interaction terms
5	Model 4 plus interaction terms		Best model from above plus Age and Age^2^
6	Best model from above plus Age and Age^2^		Model 5 plus Gender
7	Model 6 plus Gender		

*CORE-OM* Clinical Outcomes in Routine Evaluation - Outcome Measure, *LDQ* Leeds Dependence Questionnaire, *TOP* Treatment Outcomes Profile, *EQ-5D-5 L* EuroQol – 5 Dimensions – 5 Levels, *ICECAP-A* ICEpop CAPability measure for Adults

To test whether the algorithms were fit for purpose, the *R*^*2*^, the adjusted *R*^*2*^, the Akaike information criterion (AIC) and the Bayesian information criterion (BIC) were assessed. For the final model choice, Brazier et al. [14] argue that predictive ability and not fit should be considered, and therefore an internal and external validation of the models’ predictive ability was undertaken. Internal validation involved the prediction of the EQ-5D-5 L and ICECAP-A index scores from each model’s outputs and evaluated how close the predicted results were to the observed ones in the estimation dataset using the root-mean-squared-error (RMSE) and the mean absolute error (MAE) [25]. For external validation, models’ coefficients were applied to the scores of CORE-OM, LDQ, and TOP in the validation dataset and the results were plotted on a graph in order to see how close the predicted EQ-5D-5 L and ICECAP-A index scores were to the actual index scores using the RMSE and MAE. All analyses were undertaken in Stata version 13MP.

## Results

### Descriptive statistics

Table 2 presents the demographic details for the 83 trial participants. The mean age was 37 years and the sample comprised mostly men (87%). The majority were of white ethnicity (84%) and unemployed (79%). Nearly 82% of the sample received some form of state benefits. At baseline, mean capability (ICECAP-A) index score was 0.662 (SD = 0.189) and mean health (EQ-5D-5 L) index score was 0.806 (SD = 0.204). The summary statistics for the two preference-based outcome measures and for both the estimation and validation samples across the different follow-up periods are shown in Additional file 1: Table S1.

**Table 2:** Patient Demographic Information

Number in Study	83
Age, mean (SD)	*37.1 (6.40)*
Men, n (%)	*72 (86.80)*
White, n (%)	*70 (84.30)*
Employed, n (%)	*17 (20.50)*
Married, n (%)	*2 (2.40)*
Family Accommodation, n (%)	*75 (90.40)*
Secondary Education or less, n (%)	*56 (67.50)*
State Benefit Recipients, n (%)	*68 (81.90)*

*n* number of patients*, SD* standard deviation

#### Mapping CORE-OM onto EQ-5D-5 L and ICECAP-A

**Fig. 1:**
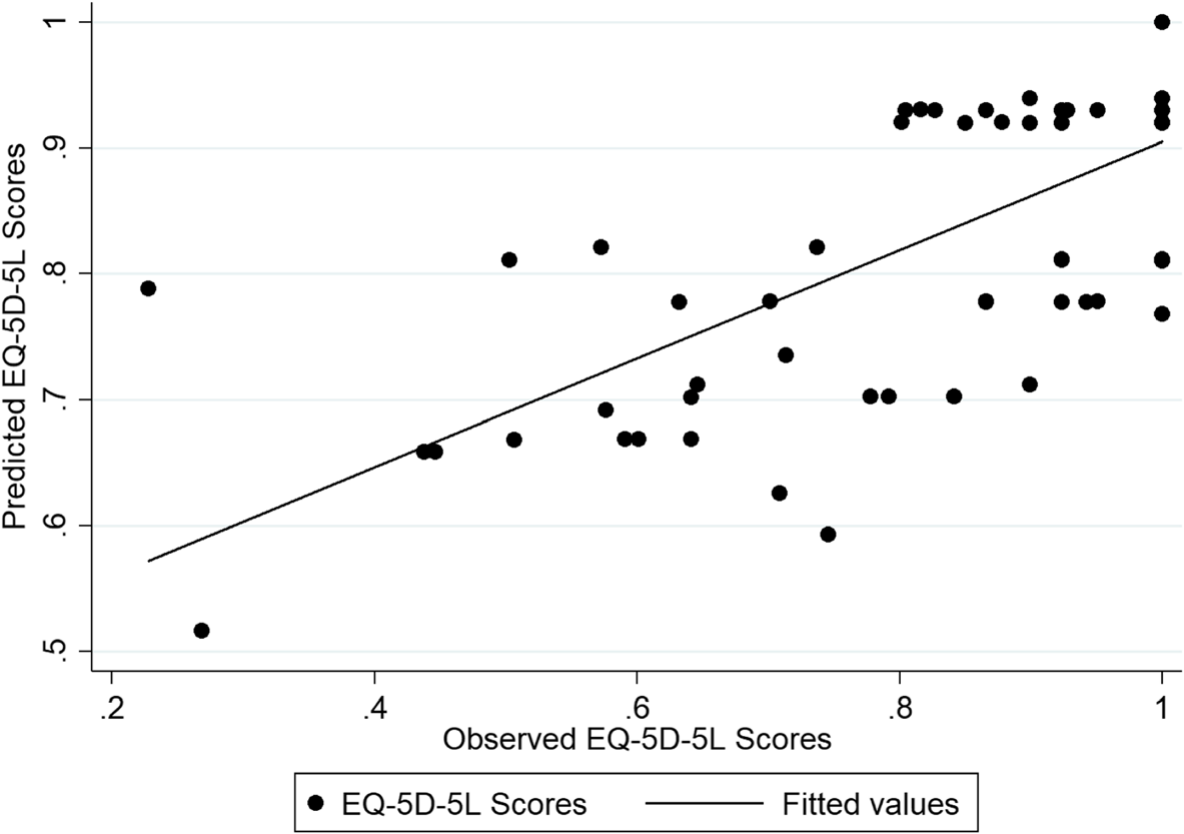
The observed vs predicted scores of the EQ-5D-5 L mapped from the CORE-OM based on Model 3

**Fig. 2:**
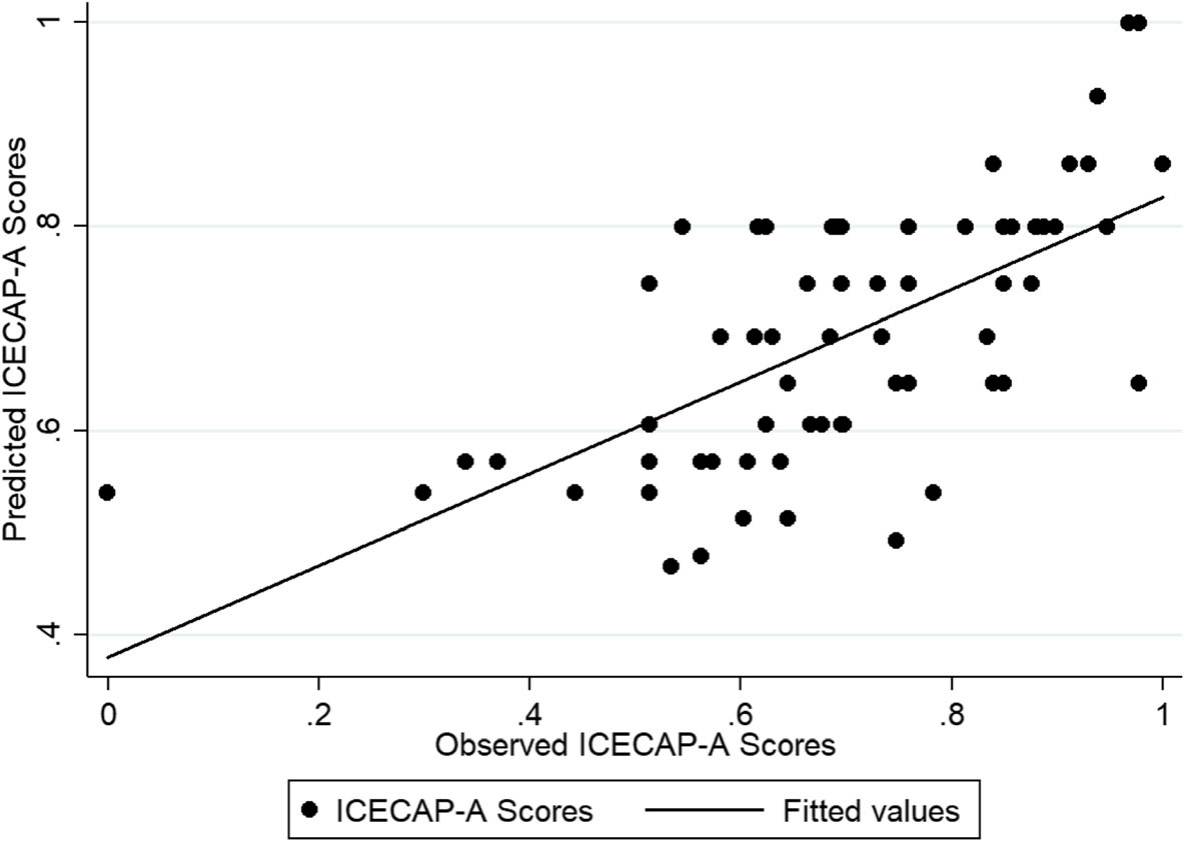
The observed vs predicted scores of the ICECAP-A mapped from the CORE-OM based on Model 2

The performance of the different models in the internal (estimation) and external validation samples is provided in Additional file 2: Table S2 and Additional file 3: Table S3, respectively. The results showed that most models predicted the EQ-5D-5 L and ICECAP-A index scores well in both samples and for both estimation approaches. For the EQ-5D-5 L most models provided predictions within a 0.03 range from the observed health index score, and therefore not different in terms of clinical importance [37]. Exceptions were mainly from the tobit regressions. For the ICECAP-A, most models provided predictions within a 0.01 range from the observed capability index scores. In the internal validation sample, model specification 5, which included the CORE-OM dimension scores plus quadratic and interaction terms, consistently produced the lowest RMSE and MAE scores across the different types of regression and the largest variability around the predicted mean EQ-5D-5 L and ICECAP-A index scores. Because of this variability, Model 5 resulted in large RMSE and MAE in the external validation sample. In this sample, model specification 3, which included the four CORE-OM dimensions as covariates, produced consistently the lowest RMSE and MAE results for the EQ-5D-5 L at the mean value as well as at repeated measurements (i.e. 25th, 50th, and 75th percentiles), whilst model specification 2, which included the mean CORE-OM score and its squared term, showed the best performance across the different models and estimation approaches (Additional file 4 Table S4). In terms of health index score, the OLS model 3 had the lowest RMSE (0.134) and MAE (0.1) and predicted the mean EQ-5D-5 L index score with a < 0.007 deviation from the observed score (0.83). In terms of the capability index score, tobit model 2 was found to have the best predictive properties with RMSE and MAE scores of 0.138 and 0.106 respectively. The coefficients for each model covariate and the model’s fit for the two mapping algorithms are shown in Table 3. Figures 1 and 2 show graphs displaying the predicted scores in comparison to the observed scores for the chosen algorithms, when mapping from the CORE-OM. The ICECAP-A is better predicted.

**Table 3:** Mapping Models from the CORE-OM to the EQ-5D-5 L and the ICECAP-A

	EQ-5D-5 L	ICECAP-A
Model	OLS (3)	Tobit (2)
Intercept	1.048^c^	0.999^c^
CORE-OM score		−0.296^a^
CORE-OM score^2^		0.041
Wellbeing	0.0005	
Symptoms	−0.109^c^	
Functioning	−0.010	
Risk	−0.033	
AIC	−56.362	−69.824
BIC	−44.451	−60.296
Adjusted R^2^/ Pseudo R^2^	0.355	−1.197
RMSE (external sample)	0.134	0.138
MAE (external sample)	0.100	0.106

^a^Statistically significant at the 1% level. *AIC* Akaike information criterion, *BIC* Bayesian information criterion, *MAE* mean absolute error, *OLS* ordinary least squares, *RMSE* root mean squared error

Using CORE-OM as an example, the EQ-5D-5 L utility score can be calculated from the following coefficients:

$$ EQ-5D-5L\  utility\ score=1.048+(Wellbeing)(0.0005)-(Symptoms)(0.109)-(Functioning)(0.010)-(Risk)(0.033) $$

#### Mapping LDQ onto EQ-5D-5 L and ICECAP-A

**Fig. 3:**
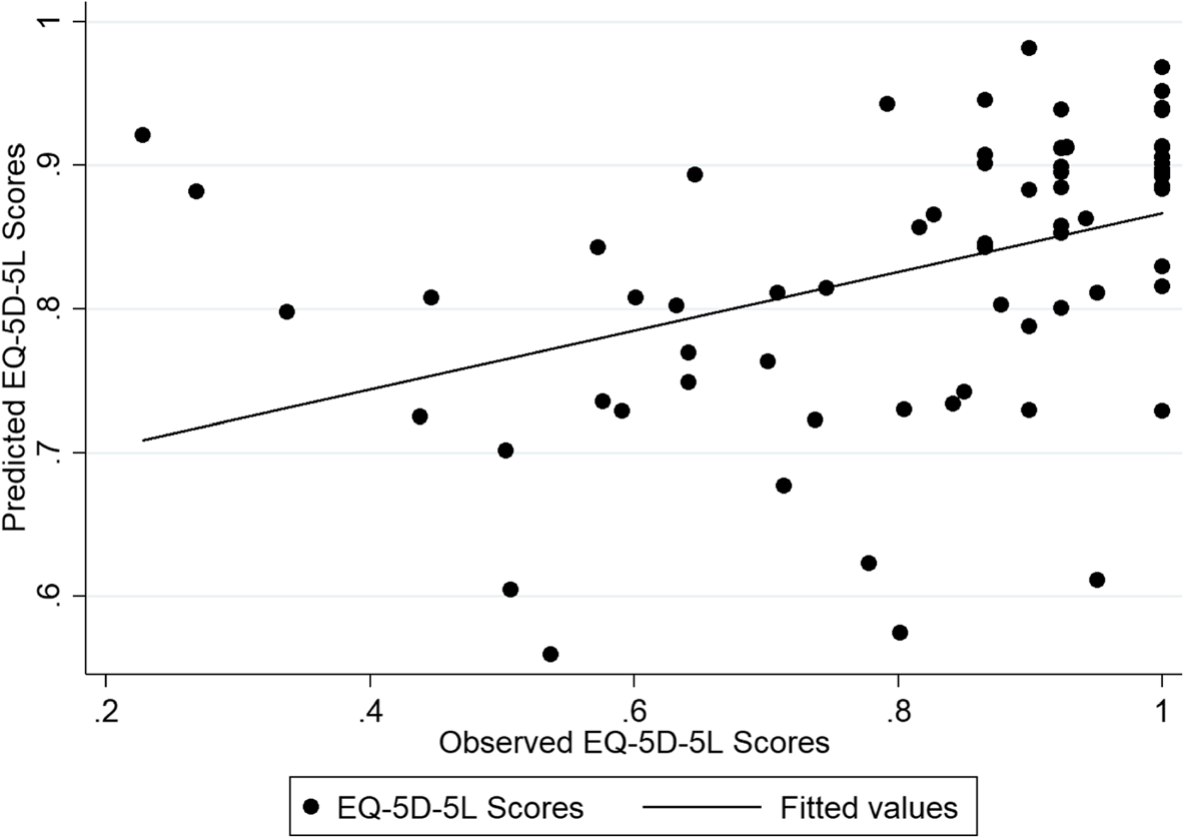
The observed vs predicted scores of the EQ-5D-5 L mapped from the LDQ based on Model 4

**Fig. 4:**
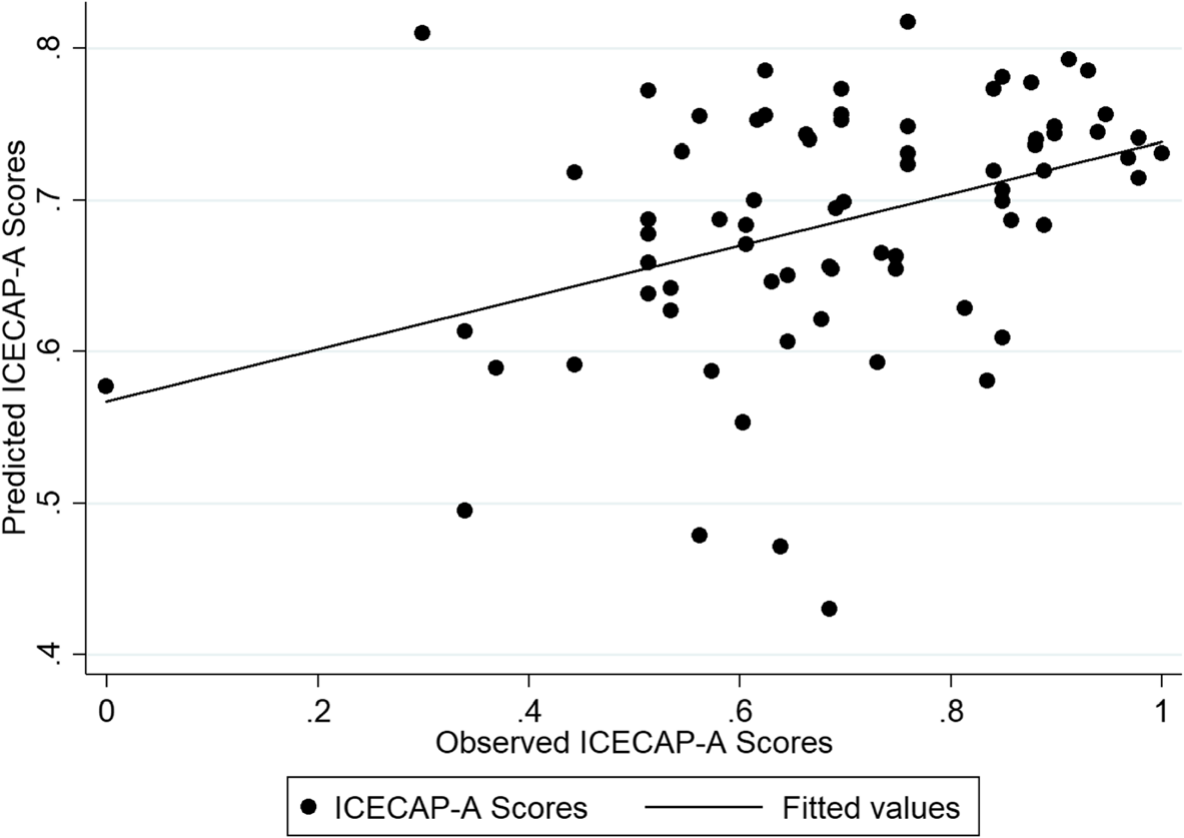
The observed vs predicted scores of the ICECAP-A mapped from the LDQ based on Model 4

Detailed information about the performance of the different models used to predict health and capability index scores in the internal and external validation samples is provided in the Additional file 5: Table S5 and Additional file 6: Table S6. Similar to the CORE-OM measure, the results indicated that most models predicted the EQ-5D-5 L and ICECAP-A index scores closely in both samples and for both estimation approaches. The only exception was from the tobit models, which gave predictions beyond the potentially acceptable 0.03 threshold difference from the observed EQ-5D-5 L index scores. For both internal and external samples, model specification 4, which included the total LDQ score, age, age squared and sex, was found to offer the best predictive ability, with RMSE and MAE ranging between 0.172–0.216 and 0.122–0.146 across the different analyses for the health index score and between 0.163–0.194 and 0.133–0.154 for the capability index score. OLS model 4 when mapping to the EQ-5D-5 L had the better predictive ability. The model produced a MAE score of 0.128 and RMSE score of 0.178. OLS model 4 also had better predictive ability when mapping to the ICECAP-A. This model produced a MAE score of 0.138 and a RMSE score of 0.171 (Additional file 7 Table S7). The coefficients for each model covariate based on the external validation sample and the model’s fit are detailed in Table 4. Figures 3 and 4 show the difference between the EQ-5D-5 L and the ICECAP-A predictions.

**Table 4:** Mapping Models from the LDQ to the EQ-5D-5 L and the ICECAP-A

	EQ-5D-5 L	ICECAP-A
Model	OLS (4)	OLS (4)
Intercept	0.415	0.958^a^
LDQ score	−0.014^b^	−0.0122^b^
Age	0.033	−0.004
Age^2^	−0.0005	−0.000002
Sex (if Female)	−0.016	−0.052
AIC	−44.943	−53.753
BIC	−32.971	−41.781
Adjusted R^2^	0.250	0.214
RMSE (external sample)	0.178	0.171
MAE (external sample)	0.128	0.138

^a^Statistically significant at the 5% level; ^b^Statistically significant at the 1% level. *AIC* Akaike information criterion, *BIC* Bayesian information criterion, *MAE* mean absolute error, *OLS* ordinary least squares, *RMSE* root mean squared error

The EQ-5D-5 L scores were more dispersed and spread than the ICECAP-A scores.

#### Mapping from the TOP onto EQ-5D-5 L and ICECAP-A

**Fig. 5:**
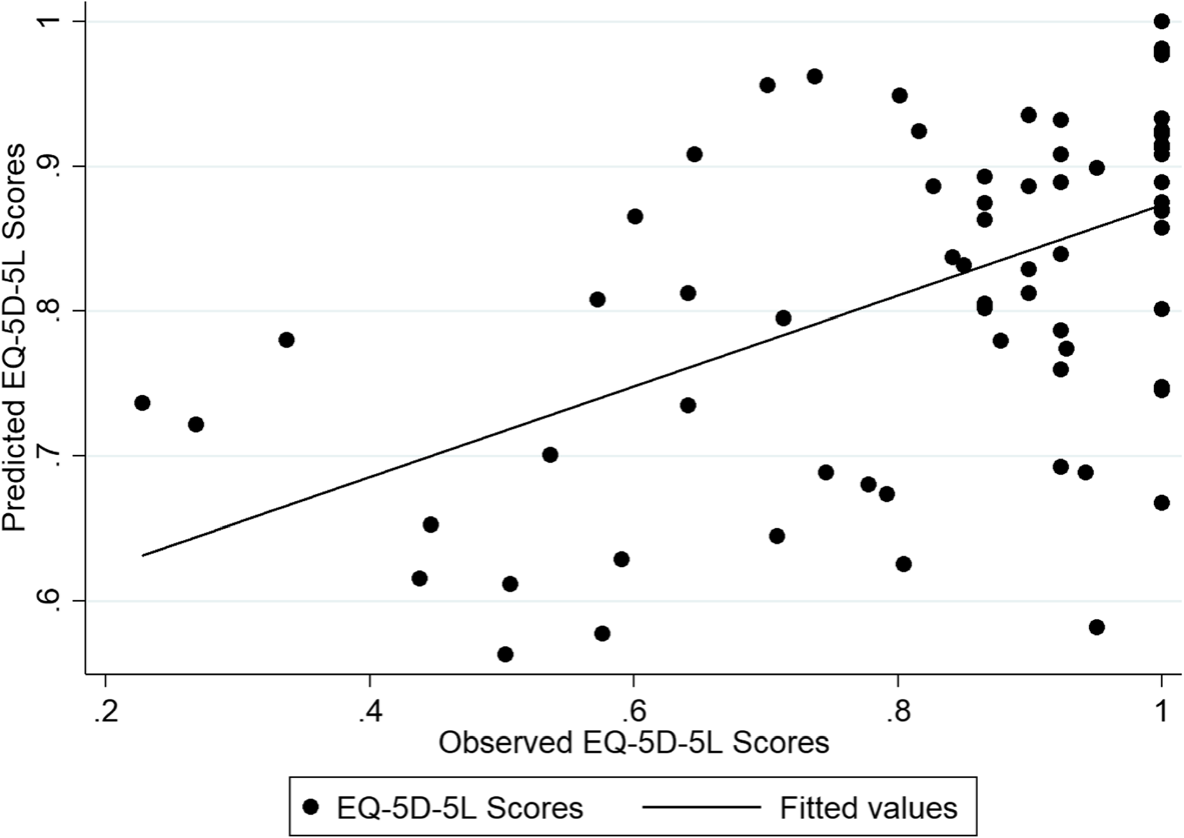
The observed vs predicted scores of the EQ-5D-5 L mapped from the TOP based on Model 2

**Fig. 6:**
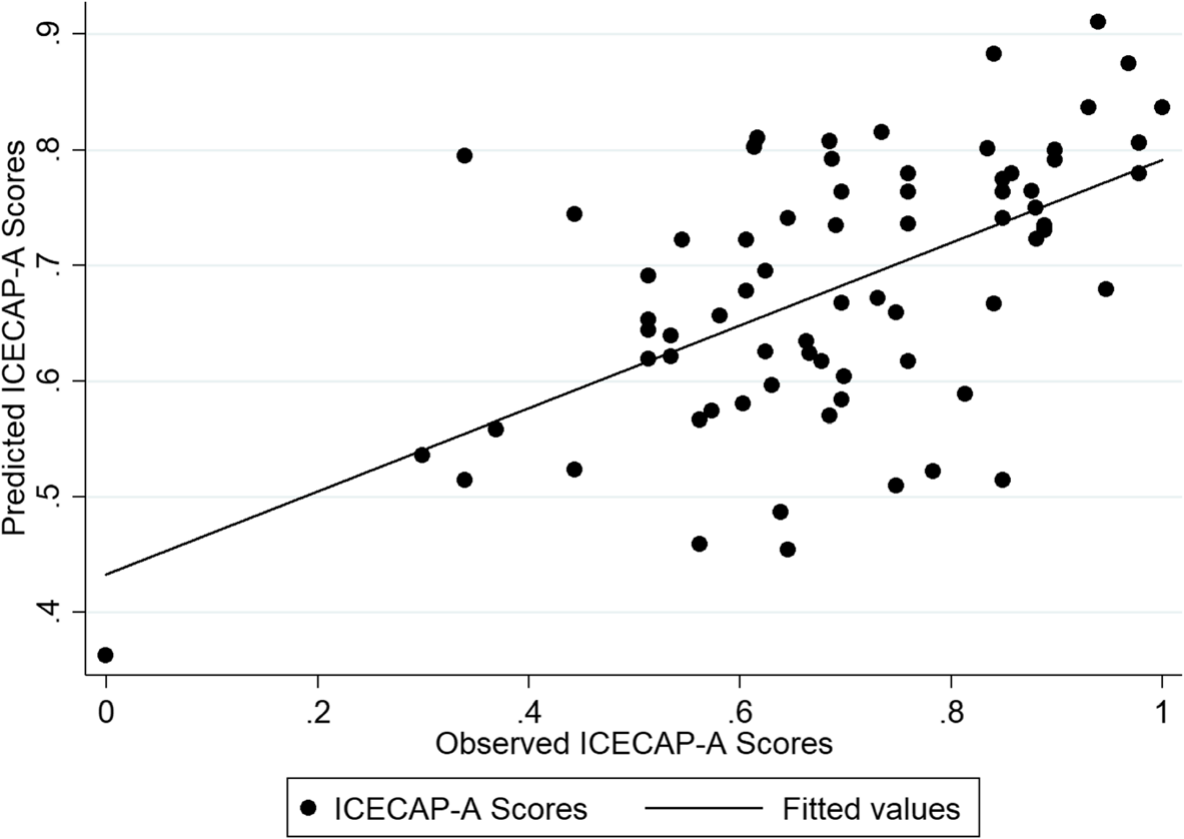
The observed vs predicted scores of the ICECAP-A mapped from the TOP based on Model 2

The performance of the different mapping algorithms from the TOP measure onto EQ-5D-5 L and ICECAP-A measures in both internal and external validation samples is shown in the Additional file 8: Table S8 and Additional file 9 Table S9. Most models predicted the EQ-5D-5 L and ICECAP-A index scores well in both samples and for both approaches. Tobit models gave again predictions of health index scores that were more than 0.03 points different to the observed EQ-5D-5 L scores but performed well in terms of predicting capability scores. Model specifications 4; which included TOP dimension scores, quadratic terms and interaction terms and model 6, which included the covariates in model 4 with the addition of age, age^2^ and gender; appeared to have a better performance in the internal sample resulting in RMSE and MAE that ranged between 0.155–0.199 and 0.122–0.126 respectively but again with larger variability around the predicted mean index scores. The model specification with the best external predictive ability across the different models and for both EQ-5D-5 L and ICECAP-A was model specification 2, which included the three TOP dimensions only (Overall quality of life score, Physical health status, and Psychological health status). Overall, the OLS model 2 predicted the observed EQ-5D-5 L (0.83) and ICECAP-A (0.69) index score with a − 0.01 point difference. For EQ-5D-5 L and ICECAP-A, the RMSE scores were 0.167 and 0.151 respectively, while the MAE scores were 0.123 for both mapping algorithms (Additional file 10: Table S10). The coefficients for each model covariate based on the external validation sample and the models’ fit are detailed in Table 5. Figures 5 and 6 display the graphs detailing the predicted and observed scores when mapping from the TOP. The EQ-5D-5 L results had a greater dispersion, and were plotted further away from the fitted value line.

**Table 5:** Mapping Models from the TOP to the EQ-5D-5 L and the ICECAP-A

	EQ-5D-5 L	ICECAP-A
Model	OLS (2)	OLS (2)
Intercept	0.463^b^	0.348^b^
Overall Quality of Life	−0.005	0.014^a^
Physical Health Status	0.010	−0.002
Psychological Health Status	0.024^b^	0.015^a^
AIC	−52.030	− 70.213
BIC	−42.402	−60.586
Adjusted R^2^	0.298	0.335
RMSE (external sample)	0.167	0.151
MAE (external sample)	0.123	0.123

^a^Statistically significant at the 5% level; ^b^Statistically significant at the 1% level. *AIC* Akaike information criterion, *BIC* Bayesian information criterion, *MAE* mean absolute error, *OLS* ordinary least squares, *RMSE* root mean squared error

## Discussion

Policy decision makers are becoming more focused on treatment outcomes that go beyond consideration of abstinence alone and capture wider treatment impact upon patients HRQOL [38]. These measures should include economic measures that allow consideration of value for money. This study developed mapping algorithms from three key clinical measures in the context of opiate dependence (CORE-OM, LDQ, and TOP) onto the EQ-5D-5 L and ICECAP-A, which are recommended by the National Institute for Health and Care Excellence (NICE) in the economic evaluation of health and social care interventions [5]. These algorithms introduce the possibility of estimating HRQOL and capability wellbeing from the information contained within each clinical measure and therefore the ability to make treatment recommendations based on wider quality of life and wellbeing outcomes. As these instruments are focused exclusively upon health-related quality of life, and wellbeing effects, these algorithms provide the vehicle to target resources towards treatment that will benefit population quality of life. The ICECAP-A was better predicted than the EQ-5D-5 L. This suggests that when mapping from the clinical measures to the ICECAP-A, there will be a greater alignment between the wellbeing aspects than when mapping to the EQ-5D-5 L. This presents an interesting development for reimbursement decisions within the context of opiate dependence and OST.

This is the first study to generate mapping algorithms for these clinical measures. This was a particularly difficult hard to reach population under study with many participants receiving OST at a therapeutic dose for at least 5 years, and still reporting heroin use [11]. The study benefited from good completion rates across the different measures, and although the primary trial focused on a hard to reach population undergoing opiate substitution treatment but still reporting heroin use, the distribution of health and capability scores provides some confidence that these algorithms are likely to be generalizable to other contexts involving substance use disorders. The algorithms developed had good predictive ability and the errors identified fall within an acceptable range in comparison to other mapping studies [14]. Demographic information, other than age and sex, was not used during the mapping process. This was a deliberate choice appreciating that future use of the mapping algorithms will be maximized by the algorithms only requiring demographic information on age and sex [4].

The study had limitations. Given that the study relies on data collected within a pilot trial, a modest sample size was used. To overcome the sample size issue, observations were pooled from two time-periods but could have led to assumptions of independence being violated between the observations and lead to ungeneralizable results. However, appropriate techniques were applied to account for within-subject dependence.

Although the sample size was effectively doubled and the dependence accounted for, the pooled dataset did not produce better results relative to the algorithms created from the normal estimation dataset. Studies, however, have been conducted with smaller samples [39, 40]. The errors reported in this study are also not significantly different to ones reported within other studies [14]. At first glance, the sample may not appear representative of the general population but was generally representative of a UK OST population with a high number of men, unemployed and people with mainly white ethnicity. The EQ-5D-5 L and ICECAP-A had maximum scores of 1. Initial regression analysis identified results that led to scores greater than one. This meant that the upper boundary had to be censored to 1. The main drawback with this approach is that the mean, RMSE and MAE scores are underestimated however there was no alternative solution as mean values above 1 are not possible.

Although, there are no published studies that map to the measures presented, there are many with other clinical measures that have mapped to the EQ-5D, which can offer a point of comparison. This study showed that applying an alternative regression specification such as the tobit regression did not improve the results, the OLS models were demonstrators of goodness of fit. This concords with other studies [41–44]. The predicted EQ-5D-5 L scores generated during the mapping process were across a much smaller spread than the observed EQ-5D-5 L scores. This has also been seen to be the case in various other studies [41, 42].

As the results were generated from a fairly small sample size, it would be useful to validate the algorithms using a larger sample. It would also be important to conduct research into how the chosen algorithms would influence QALYs and cost-effectiveness decisions in the realm of mental health.

## Conclusion

The application of the ICECAP-A could have the ability to capture mental health related quality of life outside the utility framework. In relation to mental health, recovery entails a variety of things, drug control, physical and mental health [45]. Mapping from a condition-specific measure to a traditional generic preference-based measure could miss out these key drivers within the recovery process. It is important to capture these impacts on an individual beyond the bounds of health and utilize tools such as the ICECAP-A wellbeing measure. The use of the TOP clinical measure is common practice particularly within the context of UK specialist drug treatment. Having these algorithms available provides the potential to estimate incremental QALYs and wellbeing outcomes using routinely collected data, and thus provides a framework for estimating the cost-effectiveness of alternative therapy options.

## Additional Files

Additional file 1: **Table S1.** Descriptive statistics of the generic and condition-specific measures in the estimation and validation datasets**.** Statistics describing the features of the EQ- 5D-5 L, ICECAP-A, TOP, LDQ and the CORE-OM for the estimation and validation datasets. (DOCX 16 kb)

Additional file 2: **Table S2.** Model performance of the Internal Validation Sample Mapping from the CORE-OM to the EQ- 5D-5 L and the ICECAP-A**.** Results for each model when mapping from the CORE-OM to the EQ-5D and the ICECAP-A using the internal validation sample. (DOCX 17 kb)

Additional file 3: **Table S3.** Model performance of the External Validation Sample Mapping from the CORE-OM to the EQ- 5D-5 L and the ICECAP-A**.** Results for each model when mapping from the CORE-OM to the EQ-5D and the ICECAP-A using the external validation sample. (DOCX 17 kb)

Additional file 4: **Table S4.** Model performance of the best fitting models mapping from the CORE-OM to the ICECAP-A and the EQ-5D-5 L using the external validation sample**.** Results for the best fitting models, models 2 and 3, when mapping from the CORE-OM to the EQ-5D and the ICECAP-A using the external validation sample. (DOCX 14 kb)

Additional file 5: **Table S5.** Model performance of the Internal Validation Sample Mapping from the LDQ to the EQ- 5D-5 L and the ICECAP-A**.** Results for each model when mapping from the LDQ to the EQ-5D and the ICECAP-A using the internal validation sample. (DOCX 15 kb)

Additional file 6: **Table S6** Model performance of the External Validation Sample Mapping from the LDQ to the EQ- 5D-5 L and the ICECAP-A**.** Results for each model when mapping from the LDQ to the EQ-5D and the ICECAP-A using the external validation sample. (DOCX 15 kb)

Additional file 7: **Table S7** Model performance of the best fitting models mapping from the LDQ to the ICECAP-A and the EQ-5D-5 L using the external validation sample**.** Results for the best fitting models, models 3 and 4, when mapping from the LDQ to the EQ-5D and the ICECAP-A using the external validation sample. (DOCX 14 kb)

Additional file 8: **Table S8.** Model performance of the Internal Validation Sample Mapping from the TOP to the EQ- 5D-5 L and the ICECAP-A**.** Results for each model when mapping from the TOP to the EQ-5D and the ICECAP-A using the internal validation sample. (DOCX 14 kb)

Additional file 9: **Table S9.** Model performance of the External Validation Sample Mapping from the TOP to the EQ- 5D-5 L and the ICECAP-A**.** Results for each model when mapping from the TOP to the EQ-5D and the ICECAP-A using the external validation sample. (DOCX 16 kb)

Additional file 10: **Table S10.** Model performance of the best fitting models mapping from the TOP to the ICECAP-A and the EQ-5D-5 L using the external validation sample**.** Results for the best fitting models, models 1 and 2, when mapping from the LDQ to the EQ-5D and the ICECAP-A using the external validation sample. (DOCX 14 kb)

## Abbreviations

AIC: Akaike Information Criterion
BIC: Bayesian Information Criterion
CORE-OM: Clinical Outcomes in Routine Evaluation - Outcome Measure
EQ-5D-5 L: EuroQol - 5 Dimensions- 5 Levels
HRQOL: Health-Related Quality Of Life
ICECAP-A: ICEpop CAPability measure for Adults
LDQ: Leeds Dependence Questionnaire
MAE: Mean Absolute Error.
n: Number of patients
NICE: National Institute for Health and Care Excellence
OLS: Ordinary Least Squares
OST: Opiate Substitution Treatment
QALY: Quality-Adjusted Life Years
RCT: Randomised Control Trial
RMSE: Root-Mean-Squared-Error
SD: Standard Deviation
TOP: Treatment Outcomes Profile
