# Supplementary material for: Predicting health-related quality of life (EQ-5D-5 L) and capability wellbeing (ICECAP-A) in the context of opiate dependence using routine clinical outcome measures: CORE-OM, LDQ and TOP
Source: Health Qual Life Outcomes. 2018 May 30;16:106. doi: 10.1186/s12955-018-0926-7 (PMC5975467; doi:10.1186/s12955-018-0926-7)
Supplement: Supplementary file 1 — Table S1. Descriptive statistics of the generic and condition-specific measures in the estimation and validation datasets. Statistics describing the features of the EQ- 5D-5 L, ICECAP-A, TOP, LDQ and the CORE-OM for the estimation and validation datasets. (DOCX 16 kb) [file 12955_2018_926_MOESM1_ESM.docx]

| ***Supplementary Table 1: Descriptive statistics of the generic and condition-specific measures in the estimation and validation datasets*** | | | | | | | | | | | | | | | | |
| --- | --- | --- | --- | --- | --- | --- | --- | --- | --- | --- | --- | --- | --- | --- | --- | --- |
|  | **Normal Estimation Dataset** | | | | **Pooled Estimation Dataset** | | | | **Validation Dataset 1** | | | | **Validation Dataset 2** | | | |
|  | **n** | **Mean (SD)** | **Min** | **Max** | **N** | **Mean (SD)** | **Min** | **Max** | **n** | **Mean (SD)** | **Min** | **Max** | **n** | **Mean (SD)** | **Min** | **Max** |
| **ICECAP-A** | 83 | 0.66 (0.19) | 0.17 | 1.00 | 157 | 0.68 (0.19) | 0.12 | 1.00 | 74 | 0.69 (0.19) | 0.00 | 1.00 | 140 | 0.70 (0.22) | 0.12 | 1.00 |
| **EQ-5D-5L** | 83 | 0.81 (0.20) | 0.12 | 1.00 | 157 | 0.82 (0.23) | 0.12 | 1.00 | 74 | 0.83 (0.20) | 0.23 | 1.00 | 140 | 0.81 (0.22) | 0.15 | 1.00 |
| **CORE-OM** | 80 | 1.53 (0.75) | 0.00 | 3.50 | 151 | 1.45 (0.74) | 0.00 | 3.50 | 71 | 1.36 (0.71) | 0.00 | 3.25 | 134 | 1.31 (0.76) | 0.11 | 2.92 |
| **Wellbeing** | 82 | 1.89 (0.98) | 0.00 | 4.00 | 155 | 1.84 (0.95) | 0.00 | 4.00 | 73 | 1.78 (0.95) | 0.00 | 4.00 | 140 | 1.65 (1.00) | 0.00 | 3.75 |
| **Symptoms** | 82 | 1.94 (0.97) | 0.00 | 4.00 | 155 | 1.85 (0.96) | 0.00 | 4.00 | 73 | 1.7 (0.94) | 0.00 | 4.00 | 138 | 1.65 (0.94) | 0.00 | 3.50 |
| **Functioning** | 82 | 1.78 (0.79) | 0.00 | 3.00 | 156 | 1.71 (0.78) | 0.00 | 3.00 | 74 | 1.64 (0.77) | 0.00 | 3.00 | 140 | 1.53 (0.80) | 0.08 | 3.33 |
| **Risk** | 83 | 0.54 (0.75) | 0.00 | 3.00 | 156 | 0.46 (0.70) | 0.00 | 3.00 | 73 | 0.37 (0.63) | 0.00 | 3.00 | 136 | 0.42 (0.58) | 0.00 | 2.17 |
| **LDQ** | 81 | 11.02 (7.22) | 0.00 | 30.00 | 154 | 9.96 (7.00) | 0.00 | 30.00 | 73 | 8.78 (6.52) | 0.00 | 30.00 | 140 | 9.03 (6.75) | 0.00 | 29.00 |
| **Physical Health Status (TOP)** | 83 | 11.61 (3.72) | 2.00 | 18.00 | 157 | 11.80  (3.74) | 2.00 | 19.00 | 74 | 12.01 (3.79) | 4.00 | 19.00 | 138 | 11.56 (4.03) | 2.00 | 20.00 |
| **Psychological Health Status (TOP)** | 83 | 11.43 (4.23) | 3.00 | 20.00 | 157 | 11.74 (4.32) | 2.00 | 20.00 | 74 | 12.08 (4.41) | 2.00 | 20.00 | 140 | 11.59 (4.55) | 0.00 | 20.00 |
| **Overall Quality of Life (TOP)** | 82 | 11.07 (4.42) | 0.00 | 20.00 | 154 | 11.49 (4.36) | 0.00 | 20.00 | 72 | 11.96 (4.27) | 0.00 | 2.00 | 140 | 11.84 (4.77) | 0.00 | 20.00 |
| **Min, minimum value; Max, maximum value; M, mean; SD, standard deviation; n, number of patients** | | | | | | | | | | | | | | | | |
