# Supplementary material for: Predicting health-related quality of life (EQ-5D-5 L) and capability wellbeing (ICECAP-A) in the context of opiate dependence using routine clinical outcome measures: CORE-OM, LDQ and TOP
Source: Health Qual Life Outcomes. 2018 May 30;16:106. doi: 10.1186/s12955-018-0926-7 (PMC5975467; doi:10.1186/s12955-018-0926-7)
Supplement: Supplementary file 2 — Table S2. Model performance of the Internal Validation Sample Mapping from the CORE-OM to the EQ- 5D-5 L and the ICECAP-A. Results for each model when mapping from the CORE-OM to the EQ-5D and the ICECAP-A using the internal validation sample. (DOCX 17 kb) [file 12955_2018_926_MOESM2_ESM.docx]

| ***Supplementary Table 2: Model performance of the Internal Validation Sample Mapping from the CORE-OM to the EQ- 5D-5L and the ICECAP-A*** | | | | | | | |
| --- | --- | --- | --- | --- | --- | --- | --- |
|  | | **EQ-5D-5L** | | | **ICECAP-A** | |  |
|  | Model | Mean (SD) | RMSE | MAE | Mean (SD) | RMSE | MAE |
| **OLS** | Observed | 0.806 (0.204) |  |  | 0.662 (0.189) |  |  |
|  | 1 | 0.801 (0.118) | 0.167 | 0.123 | 0.661 (0.118) | 0.147 | 0.117 |
|  | 2 | 0.802 (0.119) | 0.166 | 0.122 | 0.661 (0.120) | 0.146 | 0.115 |
|  | 3 | 0.799 (0.123) | 0.159 | 0.118 | 0.661 (0.120) | 0.146 | 0.116 |
|  | 4 | 0.801 (0.144) | 0.144 | 0.111 | 0.661 (0.128) | 0.137 | 0.111 |
|  | 5 | 0.802 (0.149) | 0.140 | 0.106 | 0.660 (0.132) | 0.131 | 0.106 |
|  | 6 | 0.801 (0.145) | 0.143 | 0.110 | 0.661 (0.130) | 0.136 | 0.109 |
|  | 7 | 0.801 (0.146) | 0.143 | 0.110 | 0.661 (0.133) | 0.133 | 0.105 |
|  |  |  |  |  |  |  |  |
| **Tobit** | Observed | 0.806 (0.204) |  |  | 0.662 (0.189) |  |  |
|  | 1 | 0.833 (0.141) | 0.204 | 0.122 | 0.662 (0.120) | 0.148 | 0.117 |
|  | 2 | 0.832 (0.140) | 0.205 | 0.122 | 0.663 (0.124) | 0.147 | 0.114 |
|  | 3 | 0.828 (0.144) | 0.196 | 0.119 | 0.662 (0.122) | 0.147 | 0.115 |
|  | 4 | 0.826 (0.159) | 0.144 | 0.111 | 0.662 (0.132) | 0.138 | 0.110 |
|  | 5 | 0.825 (0.164) | 0.140 | 0.107 | 0.662 (0.137) | 0.132 | 0.107 |
|  | 6 | 0.826 (0.160) | 0.173 | 0.110 | 0.662 (0.134) | 0.136 | 0.110 |
|  | 7 | 0.826 (0.161) | 0.177 | 0.110 | 0.662 (0.136) | 0.133 | 0.105 |
|  |  |  |  |  |  |  |  |
| **Cluster** | Observed | 0.816 (0.200) |  |  | 0.677 (0.188) |  |  |
|  | 1 | 0.817 (0.116) | 0.157 | 0.116 | 0.678 (0.121) | 0.145 | 0.114 |
|  | 2 | 0.817 (0.117) | 0.157 | 0.116 | 0.678 (0.124) | 0.142 | 0.110 |
|  | 3 | 0.815 (0.124) | 0.147 | 0.109 | 0.678 (0.123) | 0.143 | 0.111 |
|  | 4 | 0.817 (0.136) | 0.139 | 0.103 | 0.678 (0.126) | 0.141 | 0.110 |
|  | 5 | 0.816 (0.139) | 0.136 | 0.103 | 0.678 (0.131) | 0.135 | 0.107 |
|  | 6 | 0.817 (0.139) | 0.137 | 0.100 | 0.678 (0.126) | 0.140 | 0.109 |
|  | 7 | 0.817 (0.139) | 0.137 | 0.100 | 0.678 (0.127) | 0.139 |  |
|  |  |  |  |  |  |  |  |
| **Mixed** | Observed | 0.816 (0.200) |  |  | 0.677 (0.188) |  |  |
|  | 1 | 0.817 (0.111) | 0.157 | 0.116 | 0.680 (0.115) | 0.145 | 0.114 |
|  | 2 | 0.817 (0.112) | 0.157 | 0.116 | 0.679 (0.119) | 0.142 | 0.110 |
|  | 3 | 0.815 (0.120) | 0.148 | 0.109 | 0.679 (0.117) | 0.143 | 0.111 |
|  | 4 | 0.817 (0.131) | 0.140 | 0.103 | 0.679 (0.120) | 0.141 | 0.110 |
|  | 5 | 0.817 (0.132) | 0.137 | 0.105 | 0.679 (0.126) | 0.135 | 0.108 |
|  | 6 | 0.817 (0.136) | 0.137 | 0.100 | 0.679 (0.121) | 0.140 | 0.109 |
|  | 7 | 0.817 (0.136) | 0.137 | 0.100 | 0.679 (0.122) | 0.140 | 0.108 |
| ***MAE*- mean absolute error, *OLS*- ordinary least squares, *RMSE*- root mean squared error, *SD*- standard deviation** | | | | | | | |
