# Supplementary material for: Predicting health-related quality of life (EQ-5D-5 L) and capability wellbeing (ICECAP-A) in the context of opiate dependence using routine clinical outcome measures: CORE-OM, LDQ and TOP
Source: Health Qual Life Outcomes. 2018 May 30;16:106. doi: 10.1186/s12955-018-0926-7 (PMC5975467; doi:10.1186/s12955-018-0926-7)
Supplement: Supplementary file 3 — Table S3. Model performance of the External Validation Sample Mapping from the CORE-OM to the EQ- 5D-5 L and the ICECAP-A. Results for each model when mapping from the CORE-OM to the EQ-5D and the ICECAP-A using the external validation sample. (DOCX 17 kb) [file 12955_2018_926_MOESM3_ESM.docx]

| ***Supplementary Table 3: Model performance of the External Validation Sample Mapping from the CORE-OM to the EQ- 5D-5L and the ICECAP-A*** | | | | | | | |
| --- | --- | --- | --- | --- | --- | --- | --- |
|  | | **EQ-5D-5L** | | | **ICECAP-A** | |  |
|  | Model | Mean (SD) | RMSE | MAE | Mean (SD) | RMSE | MAE |
| **OLS** | Observed | 0.828 (0.195) |  |  | 0.693 (0.186) |  |  |
|  | 1 | 0.829 (0.110) | 0.145 | 0.109 | 0.689 (0.188) | 0.142 | 0.112 |
|  | 2 | 0.830 (0.109) | 0.145 | 0.110 | 0.690 (0.121) | 0.139 | 0.106 |
|  | 3 | 0.835 (0.116) | 0.134 | 0.100 | 0.690 (0.114) | 0.144 | 0.116 |
|  | 4 | 0.837 (0.134) | 0.147 | 0.100 | 0.689 (0.127) | 0.151 | 0.120 |
|  | 5 | 0.837 (0.142) | 0.162 | 0.114 | 0.681 (0.128) | 0.151 | 0.118 |
|  | 6 | 0.837 (0.134) | 0.144 | 0.097 | 0.687 (0.129) | 0.152 | 0.122 |
|  | 7 | 0.837 (0.135) | 0.147 | 0.099 | 0.689 (0.134) | 0.161 | 0.127 |
|  |  |  |  |  |  |  |  |
| **Tobit** | Observed | 0.828 (0.195) |  |  | 0.693 (0.186) |  |  |
|  | 1 | 0.866 (0.130) | 0.149 | 0.107 | 0.692 (0.126) | 0.142 | 0.112 |
|  | 2 | 0.865 (0.132) | 0.149 | 0.107 | 0.692 (0.117) | 0.138 | 0.106 |
|  | 3 | 0.870 (0.138) | 0.139 | 0.101 | 0.692 (0.117) | 0.144 | 0.115 |
|  | 4 | 0.868 (0.153) | 0.152 | 0.102 | 0.690 (0.130) | 0.150 | 0.120 |
|  | 5 | 0.866 (0.160) | 0.168 | 0.114 | 0.680 (0.133) | 0.153 | 0.120 |
|  | 6 | 0.867 (0.153) | 0.147 | 0.097 | 0.687 (0.132) | 0.152 | 0.122 |
|  | 7 | 0.869 (0.154) | 0.150 | 0.098 | 0.691 (0.139) | 0.161 | 0.128 |
|  |  |  |  |  |  |  |  |
| **Cluster** | Observed | 0.814 (0.215) |  |  | 0.696 (0.223) |  |  |
|  | 1 | 0.839 (0.119) | 0.244 | 0.165 | 0.702 (0.125) | 0.195 | 0.151 |
|  | 2 | 0.839 (0.119) | 0.244 | 0.165 | 0.706 (0.138) | 0.198 | 0.154 |
|  | 3 | 0.837 (0.122) | 0.162 | 0.112 | 0.706 (0.131) | 0.154 | 0.119 |
|  | 4 | 0.845 (0.125) | 0.162 | 0.116 | 0.709 (0.145) | 0.157 | 0.123 |
|  | 5 | 0.846 (0.125) | 0.165 | 0.119 | 0.720 (0.143) | 0.164 | 0.126 |
|  | 6 | 0.844 (0.125) | 0.162 | 0.116 | 0.711 (0.147) | 0.155 | 0.121 |
|  | **7** | 0.844 (0.126) | 0.163 | 0.117 | 0.710 (0.147) | 0.155 | 0.121 |
|  |  |  |  |  |  |  |  |
| **Mixed** | Observed | 0.814 (0.215) |  |  | 0.696 (0.223) |  |  |
|  | 1 | 0.839 (0.115) | 0.243 | 0.164 | 0.702 (0.125) | 0.194 | 0.151 |
|  | 2 | 0.839 (0.115) | 0.242 | 0.164 | 0.706 (0.133) | 0.198 | 0.154 |
|  | 3 | 0.839 (0.119) | 0.162 | 0.111 | 0.705 (0.123) | 0.157 | 0.121 |
|  | 4 | 0.846 (0.122) | 0.162 | 0.116 | 0.708 (0.139) | 0.160 | 0.124 |
|  | 5 | 0.852 (0.119) | 0.166 | 0.118 | 0.704 (0.149) | 0.160 | 0.124 |
|  | 6 | 0.845 (0.123) | 0.162 | 0.116 | 0.710 (0.141) | 0.158 | 0.122 |
|  | 7 | 0.845 (0.124) | 0.162 | 0.116 | 0.709 (0.141) | 0.158 | 0.122 |
| ***MAE*- mean absolute error, *OLS*- ordinary least squares, *RMSE*- root mean squared error, *SD*- standard deviation** | | | | | | | |
