# Supplementary material for: Predicting health-related quality of life (EQ-5D-5 L) and capability wellbeing (ICECAP-A) in the context of opiate dependence using routine clinical outcome measures: CORE-OM, LDQ and TOP
Source: Health Qual Life Outcomes. 2018 May 30;16:106. doi: 10.1186/s12955-018-0926-7 (PMC5975467; doi:10.1186/s12955-018-0926-7)
Supplement: Supplementary file 4 — Table S4. Model performance of the best fitting models mapping from the CORE-OM to the ICECAP-A and the EQ-5D-5 L using the external validation sample. Results for the best fitting models, models 2 and 3, when mapping from the CORE-OM to the EQ-5D and the ICECAP-A using the external validation sample. (DOCX 14 kb) [file 12955_2018_926_MOESM4_ESM.docx]

| ***Supplementary Table 4: Model performance of the best fitting models mapping from the CORE-OM to the ICECAP-A and the EQ-5D-5L using the external validation sample*** | | | | | | | | | | |
| --- | --- | --- | --- | --- | --- | --- | --- | --- | --- | --- |
| **Model Number** | P.25  MAE | P.50  MAE | P.75  MAE | MAE | P.25  RMSE | P.50  RMSE | P.75  RMSE | RMSE | Abs diff. <0.10(%) | Abs diff. <0.25 (%) |
| **EQ-5D-5L** | | | | | | | | | | |
| **OLS (2)** | 0.183 | 0.132 | 0.112 | 0.110 | 0.219 | 0.170 | 0.149 | 0.145 | 56.34 | 90.14 |
| **OLS (3)** | **0.155** | **0.122** | **0.106** | **0.100** | **0.201** | **0.158** | **0.141** | **0.134** | **63.38** | **97.18** |
| **Tobit (2)** | 0.194 | 0.151 | 0.123 | 0.107 | 0.230 | 0.185 | 0.159 | 0.149 | 56.34 | 90.14 |
| **Tobit (3)** | 0.166 | 0.144 | 0.120 | 0.101 | 0.212 | 0.177 | 0.153 | 0.139 | 60.56 | 95.77 |
| **ICECAP-A** | | | | | | | | | | |
| **OLS (2)** | 0.150 | 0.116 | 0.107 | 0.106 | 0.196 | 0.154 | 0.142 | 0.139 | 61.97 | 95.77 |
| **OLS (3)** | 0.171 | 0.129 | 0.115 | 0.116 | 0.204 | 0.160 | 0.147 | 0.144 | 56.34 | 91.55 |
| **Tobit (2)** | **0.148** | **0.116** | **0.107** | **0.106** | **0.194** | **0.154** | **0.143** | **0.138** | **61.97** | **94.37** |
| **Tobit (3)** | 0.170 | 0.129 | 0.115 | 0.115 | 0.203 | 0.160 | 0.148 | 0.144 | 56.34 | 91.55 |
| ***Abs. diff.* absolute difference, *MAE* mean absolute error, *RMSE* root mean squared error, *OLS* ordinary least squares, *P.25* 25^th^ percentile, *P. 75* 75^th^ percentile** | | | | | | | | | | |
