# Supplementary material for: Predicting health-related quality of life (EQ-5D-5 L) and capability wellbeing (ICECAP-A) in the context of opiate dependence using routine clinical outcome measures: CORE-OM, LDQ and TOP
Source: Health Qual Life Outcomes. 2018 May 30;16:106. doi: 10.1186/s12955-018-0926-7 (PMC5975467; doi:10.1186/s12955-018-0926-7)
Supplement: Supplementary file 5 — Table S5. Model performance of the Internal Validation Sample Mapping from the LDQ to the EQ- 5D-5 L and the ICECAP-A. Results for each model when mapping from the LDQ to the EQ-5D and the ICECAP-A using the internal validation sample. (DOCX 15 kb) [file 12955_2018_926_MOESM5_ESM.docx]

| ***Supplementary Table 5: Model performance of the Internal Validation Sample Mapping from the LDQ to the EQ- 5D-5L and the ICECAP-A*** | | | | | | | |
| --- | --- | --- | --- | --- | --- | --- | --- |
|  | | **EQ-5D-5L** | | | **ICECAP-A** | |  |
|  |  | Mean (SD) | RMSE | MAE | Mean (SD) | RMSE | MAE |
| **OLS** | Observed | 0.806 (0.204) |  |  | 0.662 (0.189) |  |  |
|  | 1 | 0.804 (0.98) | 0.180 | 0.130 | 0.661 (0.091) | 0.166 | 0.134 |
|  | 2 | 0.804 (0.097) | 0.179 | 0.129 | 0.661 (0.091) | 0.166 | 0.134 |
|  | 3 | 0.804 (0.111) | 0.172 | 0.127 | 0.661 (0.094) | 0.164 | 0.134 |
|  | 4 | 0.804 (0.110) | 0.172 | 0.126 | 0.661 (0.096) | 0.163 | 0.133 |
|  |  |  |  |  |  |  |  |
| **Tobit** | Observed | 0.806 (0.204) |  |  | 0.662 (0.189) |  |  |
|  | 1 | 0.842 (0.117) | 0.226 | 0.129 | 0.662 (0.092) | 0.168 | 0.134 |
|  | 2 | 0.842 (0.118) | 0.225 | 0.127 | 0.662 (0.092) | 0.168 | 0.134 |
|  | 3 | 0.840 (0.130) | 0.216 | 0.124 | 0.662 (0.095) | 0.166 | 0.133 |
|  | 4 | 0.840 (0.130) | 0.216 | 0.123 | 0.662 (0.097) | 0.165 | 0.133 |
|  |  |  |  |  |  |  |  |
| **Cluster** | Observed | 0.816 (0.200) |  |  | 0.677 (0.188) |  |  |
|  | 1 | 0.814 (0.077) | 0.185 | 0.136 | 0.676 (0.084) | 0.169 | 0.136 |
|  | 2 | 0.814 (0.081) | 0.183 | 0.136 | 0.676 (0.084) | 0.168 | 0.136 |
|  | 3 | 0.814 (0.099) | 0.174 | 0.129 | 0.676 (0.087) | 0.167 | 0.136 |
|  | 4 | 0.814 (0.100) | 0.173 | 0.128 | 0.676 (0.087) | 0.167 | 0.136 |
|  |  |  |  |  |  |  |  |
| **Mixed** | Observed | 0.816 (0.200) |  |  | 0.677 (0.188) |  |  |
|  | 1 | 0.815 (0.066) | 0.185 | 0.138 | 0.679 (0.072) | 0.169 | 0.136 |
|  | 2 | 0.815 (0.066) | 0.185 | 0.138 | 0.679 (0.073) | 0.169 | 0.136 |
|  | 3 | 0.815 (0.092) | 0.174 | 0.130 | 0.679 (0.074) | 0.168 | 0.136 |
|  | 4 | 0.815 (0.094) | 0.174 | 0.129 | 0.679 (0.075) | 0.167 | 0.135 |
| ***MAE*- mean absolute error, *OLS*- ordinary least squares, *RMSE*- root mean squared error, *SD*- standard deviation** | | | | | | | |
