# Supplementary material for: Predicting health-related quality of life (EQ-5D-5 L) and capability wellbeing (ICECAP-A) in the context of opiate dependence using routine clinical outcome measures: CORE-OM, LDQ and TOP
Source: Health Qual Life Outcomes. 2018 May 30;16:106. doi: 10.1186/s12955-018-0926-7 (PMC5975467; doi:10.1186/s12955-018-0926-7)
Supplement: Supplementary file 6 — Table S6 Model performance of the External Validation Sample Mapping from the LDQ to the EQ- 5D-5 L and the ICECAP-A. Results for each model when mapping from the LDQ to the EQ-5D and the ICECAP-A using the external validation sample. (DOCX 15 kb) [file 12955_2018_926_MOESM6_ESM.docx]

| ***Supplementary Table 6: Model performance of the External Validation Sample Mapping from the LDQ to the EQ- 5D-5L and the ICECAP-A*** | | | | | | | |
| --- | --- | --- | --- | --- | --- | --- | --- |
|  | | **EQ-5D-5L** | | | **ICECAP-A** | |  |
|  |  | Mean (SD) | RMSE | MAE | Mean (SD) | RMSE | MAE |
| **OLS** | Observed | 0.828 (0.195) |  |  | 0.693 (0.186) |  |  |
|  | 1 | 0.835 (0.088) | 0.192 | 0.137 | 0.689 (0.082) | 0.171 | 0.139 |
|  | 2 | 0.833 (0.089) | 0.189 | 0.137 | 0.689 (0.082) | 0.171 | 0.139 |
|  | 3 | 0.831 (0.098) | 0.178 | 0.128 | 0.686 (0.082) | 0.171 | 0.140 |
|  | 4 | 0.831 (0.097) | 0.178 | 0.128 | 0.686 (0.082) | 0.171 | 0.138 |
|  |  |  |  |  |  |  |  |
| **Tobit** | Observed | 0.828 (0.195) |  |  | 0.693 (0.186) |  |  |
|  | 1 | 0.878 (0.105) | 0.202 | 0.131 | 0.690 (0.083) | 0.171 | 0.139 |
|  | 2 | 0.875 (0.108) | 0.201 | 0.131 | 0.690 (0.083) | 0.171 | 0.139 |
|  | 3 | 0.872 (0.114) | 0.188 | 0.122 | 0.687 (0.083) | 0.171 | 0.140 |
|  | 4 | 0.872 (0.114) | 0.187 | 0.122 | 0.687 (0.083) | 0.171 | 0.138 |
|  |  |  |  |  |  |  |  |
| **Cluster** | Observed | 0.814 (0.215) |  |  | 0.696 (0.223) |  |  |
|  | 1 | 0.824 (0.074) | 0.200 | 0.144 | 0.687 (0.081) | 0.192 | 0.152 |
|  | 2 | 0.823 (0.075) | 0.200 | 0.143 | 0.687 (0.082) | 0.193 | 0.151 |
|  | 3 | 0.825 (0.101) | 0.205 | 0.146 | 0.687 (0.086) | 0.191 | 0.151 |
|  | 4 | 0.825 (0.102) | 0.205 | 0.144 | 0.687 (0.086) | 0.191 | 0.151 |
|  |  |  |  |  |  |  |  |
| **Mixed** | Observed | 0.814 (0.215) |  |  | 0.696 (0.223) |  |  |
|  | 1 | 0.824 (0.064) | 0.201 | 0.147 | 0.688 (0.069) | 0.195 | 0.155 |
|  | 2 | 0.824 (0.064) | 0.201 | 0.146 | 0.689 (0.073) | 0.196 | 0.155 |
|  | 3 | 0.824 (0.095) | 0.206 | 0.148 | 0.688 (0.073) | 0.194 | 0.154 |
|  | 4 | 0.825 (0.096) | 0.205 | 0.146 | 0.689 (0.081) | 0.194 | 0.154 |
| ***MAE*- mean absolute error, *OLS*- ordinary least squares, *RMSE*- root mean squared error, *SD*- standard deviation** | | | | | | | |
