# Supplementary material for: Predicting health-related quality of life (EQ-5D-5 L) and capability wellbeing (ICECAP-A) in the context of opiate dependence using routine clinical outcome measures: CORE-OM, LDQ and TOP
Source: Health Qual Life Outcomes. 2018 May 30;16:106. doi: 10.1186/s12955-018-0926-7 (PMC5975467; doi:10.1186/s12955-018-0926-7)
Supplement: Supplementary file 7 — Table S7 Model performance of the best fitting models mapping from the LDQ to the ICECAP-A and the EQ-5D-5 L using the external validation sample. Results for the best fitting models, models 3 and 4, when mapping from the LDQ to the EQ-5D and the ICECAP-A using the external validation sample. (DOCX 14 kb) [file 12955_2018_926_MOESM7_ESM.docx]

| ***Supplementary Table 7: Model performance of the best fitting models mapping from the LDQ to the ICECAP-A and the EQ-5D-5L using the external validation sample*** | | | | | | | | | | |
| --- | --- | --- | --- | --- | --- | --- | --- | --- | --- | --- |
| **Model no.** | P.25  MAE | P.50  MAE | P.75  MAE | MAE | P.25  RMSE | P.50  RMSE | P.75  RMSE | RMSE | Abs diff. <0.10(%) | Abs diff. <0.25 (%) |
| **EQ-5D-5L** | | | | | | | | | | |
| **OLS (3)** | 0.241 | 0.159 | 0.136 | 0.128 | 0.302 | 0.224 | 0.195 | 0.178 | 49.32 | 89.04 |
| **OLS (4)** | **0.240** | **0.158** | **0.135** | **0.128** | **0.300** | **0.223** | **0.194** | **0.178** | **49.32** | **89.04** |
| **Tobit (3)** | 0.274 | 0.183 | 0.146 | 0.122 | 0.337 | 0.250 | 0.213 | 0.188 | 60.27 | 89.04 |
| **Tobit (4)** | 0.273 | 0.182 | 0.145 | 0.122 | 0.335 | 0.249 | 0.212 | 0.187 | 60.27 | 87.67 |
| **ICECAP-A** | | | | | | | | | | |
| **OLS (3)** | 0.212 | 0.152 | 0.128 | 0.140 | 0.248 | 0.192 | 0.167 | 0.171 | 36.99 | 89.04 |
| **OLS (4)** | **0.206** | **0.147** | **0.125** | **0.138** | **0.247** | **0.191** | **0.167** | **0.171** | **41.10** | **87.67** |
| **Tobit (3)** | 0.213 | 0.152 | 0.129 | 0.140 | 0.249 | 0.193 | 0.168 | 0.171 | 36.99 | 89.04 |
| **Tobit (4)** | 0.207 | 0.147 | 0.126 | 0.138 | 0.248 | 0.191 | 0.167 | 0.171 | 41.10 | 87.67 |
| ***Abs. diff.* absolute difference, *MAE* mean absolute error, *RMSE* root mean squared error, *OLS* ordinary least squares, *P.25* 25^th^ percentile, *P. 75* 75^th^ percentile** | | | | | | | | | | |
