# Supplementary material for: Predicting health-related quality of life (EQ-5D-5 L) and capability wellbeing (ICECAP-A) in the context of opiate dependence using routine clinical outcome measures: CORE-OM, LDQ and TOP
Source: Health Qual Life Outcomes. 2018 May 30;16:106. doi: 10.1186/s12955-018-0926-7 (PMC5975467; doi:10.1186/s12955-018-0926-7)
Supplement: Supplementary file 8 — Table S8. Model performance of the Internal Validation Sample Mapping from the TOP to the EQ- 5D-5 L and the ICECAP-A. Results for each model when mapping from the TOP to the EQ-5D and the ICECAP-A using the internal validation sample. (DOCX 14 kb) [file 12955_2018_926_MOESM8_ESM.docx]

| ***Supplementary Table 8: Model performance of the Internal Validation Sample Mapping from the TOP to the EQ- 5D-5L and the ICECAP-A*** | | | | | | | |
| --- | --- | --- | --- | --- | --- | --- | --- |
|  | | **EQ-5D-5L** | | | **ICECAP-A** | |  |
|  |  | Mean (SD) | RMSE | MAE | Mean (SD) | RMSE | MAE |
| **OLS** | Observed | 0.806 (0.204) |  |  | 0.662 (0.189) |  |  |
|  | 1 | 0.806 (0.070) | 0.192 | 0.149 | 0.660 (0.104) | 0.157 | 0.127 |
|  | 2 | 0.805 (0.115) | 0.168 | 0.131 | 0.660 (0.113) | 0.150 | 0.117 |
|  | 3 | 0.806 (0.129) | 0.159 | 0.127 | 0.660 (0.114) | 0.149 | 0.116 |
|  | 4 | 0.806 (0.133) | 0.155 | 0.122 | 0.660 (0.126) | 0.140 | 0.112 |
|  | 5 | 0.806 (0.130) | 0.158 | 0.127 | 0.660 (0.127) | 0.139 | 0.113 |
|  | 6 | 0.806 (0.130) | 0.157 | 0.126 | 0.660 (0.129) | 0.136 | 0.110 |
|  |  |  |  |  |  |  |  |
| **Tobit** | Observed | 0.806 (0.204) |  |  | 0.662 (0.189) |  |  |
|  | 1 | 0.846 (0.080) | 0.246 | 0.157 | 0.661 (0.105) | 0.158 | 0.127 |
|  | 2 | 0.848 (0.153) | 0.210 | 0.129 | 0.661 (0.115) | 0.152 | 0.117 |
|  | 3 | 0.845 (0.157) | 0.200 | 0.123 | 0.661 (0.116) | 0.151 | 0.115 |
|  | 4 | 0.847 (0.179) | 0.195 | 0.117 | 0.661 (0.129) | 0.141 | 0.112 |
|  | 5 | 0.844 (0.158) | 0.199 | 0.124 | 0.661 (0.129) | 0.140 | 0.113 |
|  | 6 | 0.844 (0.158) | 0.199 | 0.123 | 0.661 (0.131) | 0.138 | 0.110 |
|  |  |  |  |  |  |  |  |
| **Cluster** | Observed | 0.816 (0.200) |  |  | 0.677 (0.188) |  |  |
|  | 1 | 0.816 (0.076) | 0.185 | 0.145 | 0.675 (0.101) | 0.158 | 0.128 |
|  | 2 | 0.816 (0.109) | 0.167 | 0.128 | 0.675 (0.112) | 0.150 | 0.120 |
|  | 3 | 0.816 (0.115) | 0.164 | 0.125 | 0.675 (0.115) | 0.148 | 0.118 |
|  | 4 | 0.816 (0.116) | 0.162 | 0.122 | 0.675 (0.118) | 0.146 | 0.118 |
|  | 5 | 0.816 (0.120) | 0.160 | 0.123 | 0.675 (0.118) | 0.145 | 0.118 |
|  | 6 | 0.816 (0.120) | 0.160 | 0.123 | 0.675 (0.119) | 0.145 | 0.117 |
|  |  |  |  |  |  |  |  |
| **Mixed** | Observed | 0.816 (0.200) |  |  | 0.677 (0.188) |  |  |
|  | 1 | 0.818 (0.064) | 0.185 | 0.145 | 0.679 (0.087) | 0.158 | 0.129 |
|  | 2 | 0.817 (0.102) | 0.167 | 0.128 | 0.679 (0.100) | 0.151 | 0.121 |
|  | 3 | 0.818 (0.104) | 0.164 | 0.126 | 0.679 (0.105) | 0.149 | 0.118 |
|  | 4 | 0.817 (0.104) | 0.163 | 0.125 | 0.679 (0.110) | 0.147 | 0.118 |
|  | 5 | 0.817 (0.112) | 0.161 | 0.124 | 0.679 (0.111) | 0.146 | 0.118 |
|  | 6 | 0.817 (0.113) | 0.161 | 0.124 | 0.679 (0.111) | 0.146 | 0.118 |
| ***MAE*- mean absolute error, *OLS*- ordinary least squares, *RMSE*- root mean squared error, *SD*- standard deviation** | | | | | | | |
