# Supplementary material for: Predicting health-related quality of life (EQ-5D-5 L) and capability wellbeing (ICECAP-A) in the context of opiate dependence using routine clinical outcome measures: CORE-OM, LDQ and TOP
Source: Health Qual Life Outcomes. 2018 May 30;16:106. doi: 10.1186/s12955-018-0926-7 (PMC5975467; doi:10.1186/s12955-018-0926-7)
Supplement: Supplementary file 9 — Table S9. Model performance of the External Validation Sample Mapping from the TOP to the EQ- 5D-5 L and the ICECAP-A. Results for each model when mapping from the TOP to the EQ-5D and the ICECAP-A using the external validation sample. (DOCX 16 kb) [file 12955_2018_926_MOESM9_ESM.docx]

| ***Supplementary Table 9: Model performance of the External Validation Sample Mapping from the TOP to the EQ- 5D-5L and the ICECAP-A*** | | | | | | | |
| --- | --- | --- | --- | --- | --- | --- | --- |
|  | | **EQ-5D-5L** | | | **ICECAP-A** | |  |
|  |  | Mean (SD) | RMSE | MAE | Mean (SD) | RMSE | MAE |
| **OLS** | Observed | 0.828 (0.195) |  |  | 0.693 (0.186) |  |  |
|  | 1 | 0.820 (0.067) | 0.177 | 0.142 | 0.681 (0.100) | 0.159 | 0.129 |
|  | 2 | 0.820 (0.118) | 0.167 | 0.123 | 0.682 (0.115) | 0.151 | 0.123 |
|  | 3 | 0.814 (0.130) | 0.176 | 0.132 | 0.681 (0.118) | 0.152 | 0.123 |
|  | 4 | 0.805 (0.136) | 0.183 | 0.138 | 0.683 (0.163) | 0.181 | 0.134 |
|  | 5 | 0.813 (0.130) | 0.177 | 0.131 | 0.683 (0.163) | 0.180 | 0.133 |
|  | 6 | 0.813 (0.131) | 0.177 | 0.133 | 0.681 (0.167) | 0.187 | 0.139 |
|  |  |  |  |  |  |  |  |
| **Tobit** | Observed | 0.828 (0.195) |  |  | 0.693 (0.186) |  |  |
|  | 1 | 0.863 (0.068) | 0.179 | 0.130 | 0.682 (0.102) | 0.159 | 0.129 |
|  | 2 | 0.855 (0.142) | 0.175 | 0.122 | 0.683 (0.117) | 0.151 | 0.123 |
|  | 3 | 0.853 (0.156) | 0.185 | 0.129 | 0.683 (0.120) | 0.152 | 0.123 |
|  | 4 | 0.841 (0.165) | 0.192 | 0.135 | 0.684 (0.166) | 0.183 | 0.135 |
|  | 5 | 0.852 (0.158) | 0.187 | 0.131 | 0.684 (0.166) | 0.182 | 0.134 |
|  | 6 | 0.852 (0.159) | 0.188 | 0.133 | 0.683 (0.170) | 0.190 | 0.140 |
|  |  |  |  |  |  |  |  |
| **Cluster** | Observed | 0.814 (0.215) |  |  | 0.696 (0.223) |  |  |
|  | 1 | 0.822 (0.084) | 0.189 | 0.146 | 0.683 (0.110) | 0.170 | 0.142 |
|  | 2 | 0.816 (0.107) | 0.200 | 0.149 | 0.688 (0.111) | 0.174 | 0.142 |
|  | 3 | 0.816 (0.116) | 0.200 | 0.149 | 0.689 (0.111) | 0.179 | 0.144 |
|  | 4 | 0.826 (0.119) | 0.203 | 0.148 | 0.678 (0.117) | 0.186 | 0.151 |
|  | 5 | 0.816 (0.119) | 0.204 | 0.153 | 0.677 (0.120) | 0.187 | 0.152 |
|  | 6 | 0.816 (0.119) | 0.204 | 0.153 | 0.676 (0.117) | 0.187 | 0.152 |
|  |  |  |  |  |  |  |  |
| **Mixed** | Observed | 0.814 (0.215) |  |  | 0.696 (0.223) |  |  |
|  | 1 | 0.819 (0.059) | 0.191 | 0.151 | 0.686 (0.095) | 0.174 | 0.144 |
|  | 2 | 0.817 (0.099) | 0.202 | 0.152 | 0.691 (0.102) | 0.175 | 0.143 |
|  | 3 | 0.816 (0.105) | 0.201 | 0.152 | 0.694 (0.103) | 0.185 | 0.147 |
|  | 4 | 0.823 (0.103) | 0.201 | 0.149 | 0.684 (0.112) | 0.194 | 0.157 |
|  | 5 | 0.816 (0.112) | 0.206 | 0.156 | 0.683 (0.115) | 0.195 | 0.158 |
|  | 6 | 0.816 (0.112) | 0.205 | 0.156 | 0.684 (0.114) | 0.194 | 0.156 |
| ***MAE*- mean absolute error, *OLS*- ordinary least squares, *RMSE*- root mean squared error, *SD*- standard deviation** | | | | | | | |
