# Supplementary material for: Predicting health-related quality of life (EQ-5D-5 L) and capability wellbeing (ICECAP-A) in the context of opiate dependence using routine clinical outcome measures: CORE-OM, LDQ and TOP
Source: Health Qual Life Outcomes. 2018 May 30;16:106. doi: 10.1186/s12955-018-0926-7 (PMC5975467; doi:10.1186/s12955-018-0926-7)
Supplement: Supplementary file 10 — Table S10. Model performance of the best fitting models mapping from the TOP to the ICECAP-A and the EQ-5D-5 L using the external validation sample. Results for the best fitting models, models 1 and 2, when mapping from the LDQ to the EQ-5D and the ICECAP-A using the external validation sample. (DOCX 14 kb) [file 12955_2018_926_MOESM10_ESM.docx]

| ***Supplementary Table 10: Model performance of the best fitting models mapping from the TOP to the ICECAP-A and the EQ-5D-5L using the external validation sample*** | | | | | | | | | | |
| --- | --- | --- | --- | --- | --- | --- | --- | --- | --- | --- |
| **Model no.** | P.25  MAE | P.50  MAE | P.75  MAE | MAE | P.25  RMSE | P.50  RMSE | P.75  RMSE | RMSE | Abs diff. <0.10(%) | Abs diff. <0.25 (%) |
| **EQ-5D-5L** | | | | | | | | | | |
| **OLS (1)** | 0.206 | 0.165 | 0.157 | 0.142 | 0.237 | 0.193 | 0.195 | 0.177 | 34.72 | 91.67 |
| **OLS (2)** | **0.179** | **0.144** | **0.139** | **0.123** | **0.219** | **0.182** | **0.181** | **0.167** | **55.56** | **84.72** |
| **Tobit (1)** | 0.205 | 0.155 | 0.148 | 0.130 | 0.244 | 0.194 | 0.200 | 0.179 | 52.78 | 83.33 |
| **Tobit (2)** | 0.178 | 0.140 | 0.139 | 0.122 | 0.226 | 0.187 | 0.189 | 0.175 | 59.72 | 84.72 |
| **ICECAP-A** | | | | | | | | | | |
| **OLS (1)** | 0.175 | 0.162 | 0.119 | 0.129 | 0.210 | 0.165 | 0.153 | 0.159 | 43.06 | 88.89 |
| **OLS (2)** | **0.162** | **0.127** | **0.118** | **0.123** | **0.198** | **0.160** | **0.149** | **0.151** | **45.83** | **91.67** |
| **Tobit (1)** | 0.175 | 0.130 | 0.120 | 0.129 | 0.211 | 0.166 | 0.154 | 0.159 | 43.06 | 88.89 |
| **Tobit (2)** | 0.162 | 0.128 | 0.119 | 0.123 | 0.198 | 0.161 | 0.151 | 0.152 | 45.38 | 91.67 |
| ***Abs. diff.* - absolute difference, *MAE* - mean absolute error, *RMSE* - root mean squared error, *OLS* - ordinary least squares, *P.25 -* 25^th^ percentile, *P. 75* - 75^th^ percentile** | | | | | | | | | | |
